# Draft Genome Sequences of Thermophiles Isolated from Yates Shaft, a Deep-Subsurface Environment

**DOI:** 10.1128/genomeA.00405-17

**Published:** 2017-06-01

**Authors:** Nitin K. Singh, Courtney Carlson, Rajesh K. Sani, Kasthuri Venkateswaran

**Affiliations:** a Jet Propulsion Laboratory, California Institute of Technology, Pasadena, California, USA; b South Dakota School of Mines and Technology, Rapid City, South Dakota, USA

## Abstract

The whole-genome sequences of seven thermophiles that could grow at >55°C, but not at 37°C, were generated. These thermophilic bacteria will play a useful role as model microorganisms, and analyzing their genomes will help to understand the observed production of novel bioactive compounds, including thermozymes and macromolecules.

## GENOME ANNOUNCEMENT

Several thermophilic spore-forming bacteria were collected from a site known to harbor vast numbers of prokaryotic diversity (1)—the former Homestake Gold Mine in Lead, South Dakota, USA, now known as the Sanford Underground Research Facility (SURF) (2). The microbiome of this environment is relatively little studied and is host to significant evolutionary pressures exerted by its naturally inhospitable conditions (hot, dark, and deep subsurface), rendering it a probable location to find hardy novel microbes. Within SURF, 123 thermophilic isolates were collected from the Yates Shaft (44.35° N, 103.76° W) at a depth of 1,480 m, where the temperature was 55°C and pH was 7.5. Initially, the 16S rRNA gene was sequenced to phylogenetically identify all 123 thermophiles, and a subset of these spore-forming thermophiles was tested for resistance to UVC_254_ irradiation. Based on the results of these two tests, seven strains were chosen for whole-genome sequencing, and phylogenetic analyses showed they belonged to the genus *Geobacillus*.

Uncovering the molecular mechanisms of extremophiles, such as *Geobacillus* spp., will advance knowledge about the resistance of microorganisms to extreme extraterrestrial conditions (UV, etc.) and will play a crucial role in the field of astrobiology. Previously, UV resistance has been tested among large assortments of *Bacillus* spp. (3), but underlying genetic mechanisms of UV resistance have been not been reported in *Geobacillus* spp. In addition, thermophiles and their associated biomolecules are also germane to the development of new biotechnology. For example, the ingenious manipulation of thermostable enzymes (e.g., *Taq* polymerase) has led to the development of widely used molecular biology techniques (4). Furthermore, the industrial applications of thermostable enzymes are widespread in diverse fields (5, 6). They are convenient biocatalysts for these applications due to their ability to maintain activity at extreme temperatures (60 to 70°C) and to resist destruction by organic solvents, detergents, low or high pH, and other denaturing agents (7–9). Operating industrial processes at elevated temperatures with the aid of thermostable enzymes is desirable because it facilitates the use of higher substrate concentrations, lower viscosity, reduced risk of mesophilic microbial contamination, and high reaction rates (10, 11).

In this study, we determined the draft genome sequences of seven strains isolated from SURF. Whole-genome sequencing was done on an Illumina HiSeq2500 instrument with a paired-end module. The NGS QC Toolkit version 2.3 (12) was used to filter the data for high-quality vector- and adaptor-free reads for genome assembly (cutoff read length for high quality, 80%; cutoff quality score, 20). High-quality vector-filtered reads were assembled with MaSuRCA (13), under the default parameter predicted by the assembler for each genome, and annotated with the help of the Rapid Annotations using Subsystems Technology (RAST) (14). Table 1 summarizes assembly statistics (number of contigs, total genome size, *N*_50_ size, final coverage, G+C percentage, number of error-corrected reads used for assembly, and number of coding sequences). The genome sizes were in the range of 3.2 to 3.5 Mb per genome. The G+C content was in the range of 42.3 to 52.6% for all of the strains under study.

**TABLE 1:** Statistics summary for the seven draft Yates Shaft thermophilic bacterial genome sequences

Strain	Strain designation	NCBI accession no.	Isolation location	No. of contigs	Genome size (bp)	*N*_50 _(bp)	Final coverage (%)	% GC	No. of error-corrected reads	Total no. of genes
*Geobacillus* sp. 44B	SURF-44B	NADP00000000	Yates Shaft, SURF	284	3,543,185	35,604	100	44.62	54,051,938	3,676
*Geobacillus* sp. 44C	SURF-44C	NADQ00000000	Yates Shaft, SURF	121	3,220,397	85,620	100	42.32	66,691,843	3,365
*Geobacillus* sp. 46C-IIa	SURF-46C-IIa	NADR00000000	Yates Shaft, SURF	51	3,474,909	191,187	100	52.06	67,323,860	3,552
*Geobacillus* sp. 47C-IIb	SURF-47C-IIb	NADS00000000	Yates Shaft, SURF	167	3,347,490	59,962	100	49.60	58,967,837	3,453
*Geobacillus thermocatenulatus*	SURF-48B	NAGD00000000	Yates Shaft, SURF	230	3,386,398	43,530	100	52.63	62,078,032	3,626
*Geobacillus thermocatenulatus*	SURF-114	NAGE00000000	Yates Shaft, SURF	153	3,431,480	52,431	100	52.36	60,187,546	3,591
*Geobacillus thermocatenulatus*	SURF-189	NAGF00000000	Yates Shaft, SURF	363	3,553,583	32,887	100	52.37	51,162,127	3,854

### Accession number(s).

The whole-genome sequence data were deposited at DDBJ/EMBL/GenBank under the accession numbers listed in Table 1. The versions described in this paper are the first versions.
